# Diagnostic accuracy of SMART-COP Score for predicting the need of intensive respiratory and vasopressor support in adults with community-acquired pneumonia-Study from a low resource tertiary care center

**DOI:** 10.12669/pjms.42.(ICON26).15711

**Published:** 2026-04

**Authors:** Sabahat Fatima, Saima Ali, Hiba Kauser, Syed Ghazanfar Saleem

**Affiliations:** 1 Dr. Sabahat Fatima, *FCPS*. Emergency Department, Recep Tayyip Erdogan Hospital (Managed by Indus Hospital & Health Network), Muzzafargarh, Pakistan; 2 Dr. Saima Ali, *FCPS, MPHE, PhD., Department of Emergency Medicine, The Indus Hospital & Health Network (IHHN), Karachi, Pakistan*; 3 Dr. Hiba Kauser, *FCPS*. Department of Emergency Medicine, Aga Khan University Hospital, Karachi, Pakistan; 4 Dr. Syed Ghazanfar Saleem, *FCPS., Department of Emergency Medicine, The Indus Hospital & Health Network (IHHN), Karachi, Pakistan*

**Keywords:** Community-acquired pneumonia, Emergency department, Pakistan

## Abstract

**Background & Objective:**

Community-acquired pneumonia (CAP) is a predominant cause of emergency department (ED) visits. Bedside scoring tools are essential for early identification of at-risk patients in need of intensive respiratory and vasopressor support (IRVS). The aim of the study was to determine the diagnostic accuracy of SMART-COP as a bedside tool for predicting the need for IRVS in adult ED patients presenting with CAP.

**Methodology:**

A prospective diagnostic accuracy study was conducted at the Indus Hospital and Health Network (IHHN) ED, Karachi from November 2020 to May 2021. Adults (> 18 years) were enrolled. The reference standard was IRVS during hospitalization. Sensitivity, specificity, predictive values and area under the receiver operating characteristic (ROC) curve (AUC) were calculated. Multivariate logistic regression identified independent predictors for IRVS.

**Results:**

Out of the 209 participants, 86 (41.1%) required IRVS. At a cut-off of >3, SMART-COP showed 94.2% sensitivity, 27.6% specificity with AUC 0.71. Each one-point increase in SMART-COP doubled the odds of IRVS (adjusted OR 1.80, 95% CI: 1.43-2.26). Performance was lower in COVID-19 positive patients (sensitivity 94.7%, specificity 26.0%, AUC 0.695) as compared to COVID-19 negative patients (sensitivity 100%, specificity 35.3%, AUC 0.877).

**Conclusion:**

SMART-COP demonstrated high sensitivity and modest specificity in ED for predicting IRVS in patients with CAP. Its simplicity warrants multicenter validation and integration with clinical practice guidelines.

## INTRODUCTION

Community-acquired pneumonia (CAP) remains a leading cause of ED visits worldwide, with an increased burden in low-and-middle-income countries (LMICs) where delayed escalation to critical care is common.^1^ In Pakistan, the unequal distribution of healthcare resources mandates accurate triage and assigning appropriate care to patients.^2^ Bedside severity scores such as CURB-65^3^ and pneumonia severity index (PSI)^4^ serve as mortality predictors in CAP. SMART-COP^5^,^6^ was developed to predict the need for IRVS. The utility of SMART-COP (systolic blood pressure, multilobar infiltrates, albumin, respiratory rate, tachycardia, confusion, oxygen, and pH) in South-Asian population, especially in the COVID-19 era is understudied.

The aim of the study was to assess the diagnostic accuracy of SMART-COP in predicting the need of IRVS in adult CAP patients who presented to a low resource tertiary care ED in Karachi.

## METHODOLOGY

The prospective diagnostic accuracy study was conducted at the Indus Hospital and Health Network (IHHN) ED, Karachi from November 2020 to May 2021. The design and reporting follow the Standards for Reporting Diagnostic Accuracy Studies (STARD) guidelines.^7^ Adult patients (>18 years of age) with CAP were enrolled. Patients who were immunocompromised, had hospital-acquired pneumonia (HAP), were terminally ill or had incomplete records were excluded. All patients were followed through hospitalization to determine the need for IRVS.

SMART-COP with a range of 0-11 was used as the index test. Cut-offs of ≥3, ≥4 and ≥5 were evaluated. Clinicians calculating the score were blinded to the final IRVS status. The reference standard was the requirement for IRVS at any time during hospitalization. Decisions were made by the ED and critical care physicians independent of the index test results.

Data were analyzed using SPSS version 26. Continuous variables were reported as means ± SD and categorical variables were reported as percentages. Sensitivity, specificity, positive and negative predictive values, and AUC with 95% confidence intervals (CI) were computed for each cut-off. Multivariate logistic regression was used to assess independent predictors of IRVS. Subgroup analyses were performed for COVID-positive and COVID-negative cases. P< 0.05 was considered significant.

### Ethical considerations:

The study was conducted after seeking institutional review board (IRB) approval from IHHN (IRB_IRD_2019_12_006; dated November 1, 2020). Informed written consent was obtained from all participants. Because the study was conducted during the COVID-19 pandemic, all data were collected using infection-prevention protocols and no additional procedures beyond routine clinical acre were performed.

SMART-COP scoring and the assessment for IRVS were part of standard ED evaluation and participation in the study did not influence clinical decision-making. IRVS was not denied or withheld for research purpose. All patients received care according to established clinical guidelines. Confidentiality was maintained through data anonymization and coding. The data were password protected and available only to the principal investigator (PI).

## RESULTS

**Fig.1:**
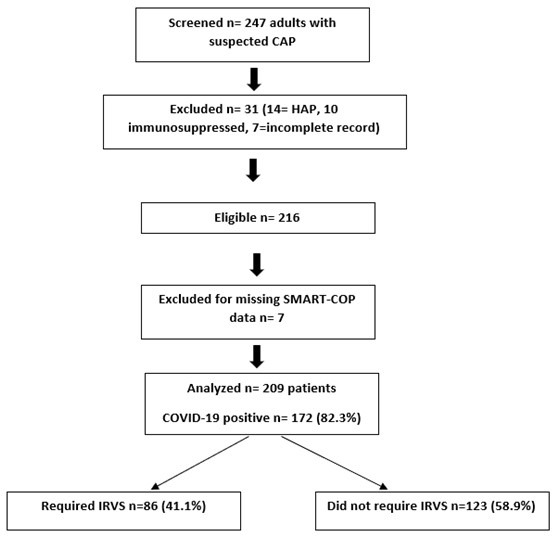
Flow diagram of participant recruitment, exclusion and final analytic cohort.

Out of the 247 screened patients, 209 were included in the final analysis after data cleaning (Fig.1). The mean age was 54.9 ± 13.7 years with 28% women. Comorbidities were present in 62% while 82% of the patients tested COVID positive. During hospitalization, IRVS was required by 41% of the patients (Table-I).

**Table-I:** Baseline characteristics of the participants (n=209).

Variable	Mean ± SD / n (%)
Age (years)	54.9 ± 13.7
Women	58 (27.8 %)
COVID-positive	172 (82.3 %)
≥ 1 comorbidity	130 (62.2 %)
IRVS required	86 (41.1 %)

**Fig.2:**
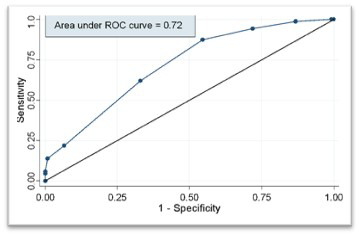
Receiver-operating characteristic (ROC) curve of SMART-COP total score predicting need for intensive respiratory or vasopressor support.

At a SMART-COP cut-off of ≥3, sensitivity was 94.2% (95% CI: 87.1-97.5) and specificity was 27.6% (95% CI: 20.5-36.1). Increasing the threshold to ≥ 4 improved specificity (41.1%) but slightly reduced sensitivity (89.4%). At SMART-COP threshold of >5, specificity further rose to 52.0% but there was a modest decline in sensitivity (83.5%). The overall AUC for SMART-COP score was 0.706 (95% CI: 0.637-0.769) (Fig.2, Table-II).

**Table-II:** Diagnostic performance of SMART- COP for predicting IRVS.

Cut-off (≥)	Sensitivity (95 % CI)	Specificity (95 % CI)	PPV (95 % CI)	NPV (95 % CI)	Accuracy
3	94.2 (87.1–97.5)	27.6 (20.5–36.1)	47.6 (40.3–55.1)	87.2 (73.3–94.4)	55.0 %
4	89.4 (80.4–94.7)	41.1 (32.6–50.1)	51.3 (43.0–59.5)	84.8 (73.1–92.0)	62.2 %
5	83.5 (73.3–90.4)	52.0 (43.1–60.8)	55.9 (47.0–64.5)	81.3 (70.7–88.8)	64.1 %

Each one-point increase in SMART-COP was associated with higher odds of IRVS (adjusted OR 1.80, 95% CI:1.43-2.26, p < 0.001). A SMART-COP threshold of ≥3 was independently associated with a six-fold increase in IRVS need (adjusted OR 6.14, 95% CI: 2.28-16.52, p= 0.0003) (Table-III).

**Table-III:** Adjusted multivariate logistic regression models predicting IRVS

Model	Predictor	Adjusted OR (95 % CI)	p-value
Continuous SMART-COP	Each 1-point increase	1.80 (1.43–2.26)	< 0.001
Dichotomous ≥ 3	SMART-COP ≥ 3	6.14 (2.28–16.52)	0.0003

In COVID-19 positive patients SMART-COP displayed high sensitivity (94.7%) but showed lower specificity (26.0%) with an AUC of 0.695. In COVID-negative patients, its performance improved, with 100% sensitivity, 35.3% specificity and an AUC of 0.877 (Table-IV). The findings indicated that SMART-COP performed less accurately in COVID-positive patients, where many scored high without needing IRVS (AUC 0.695). In contrast, the score showed a stronger ability to identify patients with COVID-negative CAP in need of IRVS (AUC 0.877).

**Table-IV:** Diagnostic performance of SMART-COP stratified by COVID-19 status.

Subgroup	Sensitivity (95 % CI)	Specificity (95 % CI)	AUC (95 % CI)	Accuracy
COVID-positive	94.7 (87.2–97.9)	26.0 (18.3–35.6)	0.695 (0.623–0.768)	56.4 %
COVID-negative	100 (61.0–100)	35.3 (17.3–59.0)	0.877 (0.718–0.983)	52.2 %

## DISCUSSION

This diagnostic accuracy study demonstrated that SMART-COP is a useful bedside tool in predicting the need for IRVS in adult patients with CAP in a resource-limited tertiary care ED in Pakistan. These results align with findings from Australia, Europe and Asia, indicating the score’s consistency across diverse settings.^8^

While the specificity was modest, a sensitive bedside tool like SMART-COP is valuable in LMIC EDs, where limited resources mandate wise use of critical care resources. A similar study from Nepal highlighted SMART-COP as a worthwhile tool for critical healthcare resource allocation.^9^ The utility of SMART-COP in predicting the need for intervention, makes it operationally relevant for ED clinicians, especially in Pakistan, where diverse demographics and comorbidities pose a challenge for expedited care provision.

The COVID-19 subgroup analysis highlighted another point. There was attenuated discrimination (AUC 0.695) despite preserved sensitivity in COVID-positive patients, likely reflecting hypoxemia-driven score inflation. In contrast, discrimination was markedly better in COVID-negative CAP (AUC 0.877), supporting SMART-COP’s stronger performance in classical bacterial pneumonia and reinforcing its generalizability across mixed-etiology pneumonias.^10^ However, clinicians would be urged to use it as a sensitive rule-out tool in COVID-positive cases and expect higher overall accuracy in patients with non-COVID CAP.

From a health systems perspective, SMART-COP’s simplicity and low cost along with its high sensitivity are valuable in Pakistan, where critical care resources are limited and delayed escalation of care is common.^11^ In such contexts, the cost of false positives is out-weighted by the risk of missing patients who require early ventilatory support. The implementation of SMART-COP can optimize resource allocation by prompting early Pulmonology consults and prioritizing transfer to high-dependency units for such patients.^12^ By following through hospitalization, defining the need for IRVS explicitly and using multivariate models, the study lays ground work for multicenter validation, electronic integration of SMART-COP within the IHHN hospital management information system (HMIS) and potential improvement of bedside tools for diagnosis of CAP.

### Limitations:

First, it’s single-center design in a high-volume ED limits generalizability to other settings with different staffing models, bed-side clinical protocols and case-mix. Second, the study period coincided with high prevalence of COVID-19 pandemic, which may have affected SMART-COP performance and calibration, as COVID-related hypoxemia can inflate score components independent of bacterial severity. Third, SMART-COP showed modest specificity at the ≥3 threshold, emphasizing that it should be used as a rule-out and early escalation tool, rather than as a definitive intensive care unit (ICU) allocation instrument. Time to IRVS, ICU length of stay (LOS) or downstream clinical outcome were not evaluated, which may have provided further insight into the score’s operational impact. Finally, in spite of using multivariate modelling, some unmeasured clinical or decision-making factors may still have influenced the outcomes, hence, residual confounding could not be ruled out.^13^

## CONCLUSION

SMART-COP showed high sensitivity and clinically useful rule-out performance for predicting IRVS among adults with CAP at ED presentation. A threshold of ≥3 can support early identification for escalation pathways in a resource-limited setting, while acknowledging trade-offs in specificity.

### Recommendations:

Integrating SMART-COP into ED bedside decision making protocols with operational endpoints (time-to-escalation, etc.), evaluating dynamic thresholds by age/ comorbidity and developing electronic dashboards can enable real-time risk flagging and early escalation of patients with CAP. Further multi-center studies should explore AI-assisted scoring and compare SMART-COP with newer biomarker-based models.

### Author’s Contribution:

**SF and SA:** Conceived, designed and did statistical analysis & manuscript writing.

**SGS and HK:** Literature review, data collection, data interpretation and manuscript editing.

All authors have read the final version and are responsible and accountable for the accuracy and integrity of the work.
